# Associations of handgrip weakness and asymmetry with new-onset stroke in Chinese middle-aged and older adults: a cohort study

**DOI:** 10.3389/fpubh.2023.1251262

**Published:** 2023-10-16

**Authors:** Yuying Zhang, Weiqing Chen, Bing Cao, Li Lin, Jinghua Li, Vivian Yawei Guo

**Affiliations:** ^1^Department of Child Healthcare, Shenzhen Longhua Maternity and Child Healthcare Hospital, Shenzhen, China; ^2^Department of Epidemiology, School of Public Health, Sun Yat-sen University, Guangzhou, China; ^3^Department of Neurosurgery, Wu Tsai Neuroscience Institute, Stanford University School of Medicine, Stanford, CA, United States; ^4^Department of Biostatistics, School of Public Health, Sun Yat-sen University, Guangzhou, China

**Keywords:** handgrip strength, weakness, asymmetry, stroke, cohort study

## Abstract

**Background:**

Weak handgrip strength (HGS) has been linked to adverse health outcomes including stroke. However, the joint associations of HGS weakness and asymmetry between limbs with stroke incidence remain underexplored.

**Methods:**

This cohort study analyzed data of participants aged ≥45 years from three waves (2011, 2013, and 2015) of the China Health and Retirement Longitudinal Study. Weak HGS was defined according to the recommendation of European Working Group on Sarcopenia in Older People. Asymmetric HGS was defined if the HGS ratio of both hands was over 1.1 or below 0.9. New-onset stroke was confirmed through self-report of physician’s diagnosis.

**Results:**

A total of 10,966 participants without stroke at baseline were included in the analysis. During the 4 years follow-up, there were 262 (2.39%) new-onset stroke cases. Compared to individuals with non-weak and symmetric HGS, those with HGS asymmetry alone and weakness alone were associated with hazards of 1.09 (95% confidence interval [CI]: 0.80–1.48) and 1.27 (95%CI: 0.86–1.88) for new-onset stroke, respectively, while co-occurrence of both HGS asymmetry and weakness was associated with 1.80 (95%CI: 1.24–2.60) greater hazard for new-onset stroke after controlling for confounders. Such associations were consistent in older adults aged ≥60 years, but not in those aged<60 years.

**Conclusion:**

Individuals with both weak and asymmetric HGS tended to have greater risk of new-onset stroke, compared to those with normal HGS, or with either weak or asymmetric HGS alone. Our finding suggested that examining HGS asymmetry alongside weakness may help to improve the risk-stratification and target prevention of stroke, particularly in the older population.

## Background

Globally, stroke is the third leading cause of mortality and a major contributor to long-term disability (1). In 2019, it was responsible for 11.6% of global deaths and 5.7% of the total disability-adjusted life-years (DALYs) (1). With the rapidly ageing population and increases in the prevalence of hypertension and diabetes mellitus in China, the burden of stroke is on the rise, posing an enormous challenge to families and the society as a whole (2, 3). Therefore, identifying modifiable risk factors associated with stroke incidence is of great importance in formulating prevention and treatment strategies.

Handgrip strength (HGS) is a convenient, reliable, and inexpensive assessment of upper limb muscle strength, which could reflect the overall muscle capacity and physical fitness (4). Low HGS has been linked to several adverse health outcomes, including cardiovascular disease, cognitive impairment, disability, and mortality (5–7). Based on the evidence, routine implementation of HGS measurement has been recommended in health care and community settings, especially for the ageing population (6). Nevertheless, most of the current research focuses solely on HGS weakness, while its asymmetry was less discussed (8).

Asymmetric HGS has been found to be detrimental to physical health (9–14). For example, a longitudinal analysis using data from the Health and Retirement Study (HRS) has reported that both HGS weakness and asymmetry independently predicted future activity limitations in Americans aged ≥50 years (9). Likewise, HGS asymmetry has also been found to be a significant predictor of neurodegenerative disorders among Chinese adults aged 60 years and over (10). In addition, several studies have demonstrated a combined effect of HGS weakness and asymmetry on health outcomes, showing greater risks of morbidity accumulation (11), reduced cognitive functioning (12), and functional disability (13, 14) in individuals with both weak and asymmetric HGS.

As the largest organ in the body, skeletal muscle is central to whole-body energy metabolism. Declines in skeletal muscle, reflected by HGS weakness, may result in metabolic dysfunctions like impaired glucose uptake and insulin resistance (15). In addition, HGS weakness has been identified as a risk factor to cardiovascular risk factors, including increased levels of blood pressure (BP), cholesterol, and inflammation (16–18). These conditions can subsequently contribute to an elevated risk of stroke (19–21). Therefore, prospective studies have revealed that HGS weakness was significantly associated with increased risk of stroke (5, 22). However, while HGS asymmetry indicates dysfunction of overall strength capacity, its combined impacts with HGS weakness in the development of new-onset stroke is underexplored. In this study, we aimed to investigate the associations of HGS weakness and asymmetry with the risk of incident stroke among Chinese middle-aged and older adults. Our study may contribute to the establishment of a clinical approach that combines HGS weakness and asymmetry for stroke risk stratification and targeted prevention.

## Methods

### Study design and population

Data of Chinese adults aged 45 years and older were drawn from the China Health and Retirement Longitudinal Study (CHARLS), a nationally representative survey aiming at promoting scientific research on healthy ageing (23–26). The baseline survey was conducted from June 2011 to March 2012, with participants selected from 450 villages/resident communities across China using a multistage probability-proportional-to-size sampling method. The respondents were followed up every 2 years, with a small share of new participants recruited in each follow-up survey.

**Figure 1:**
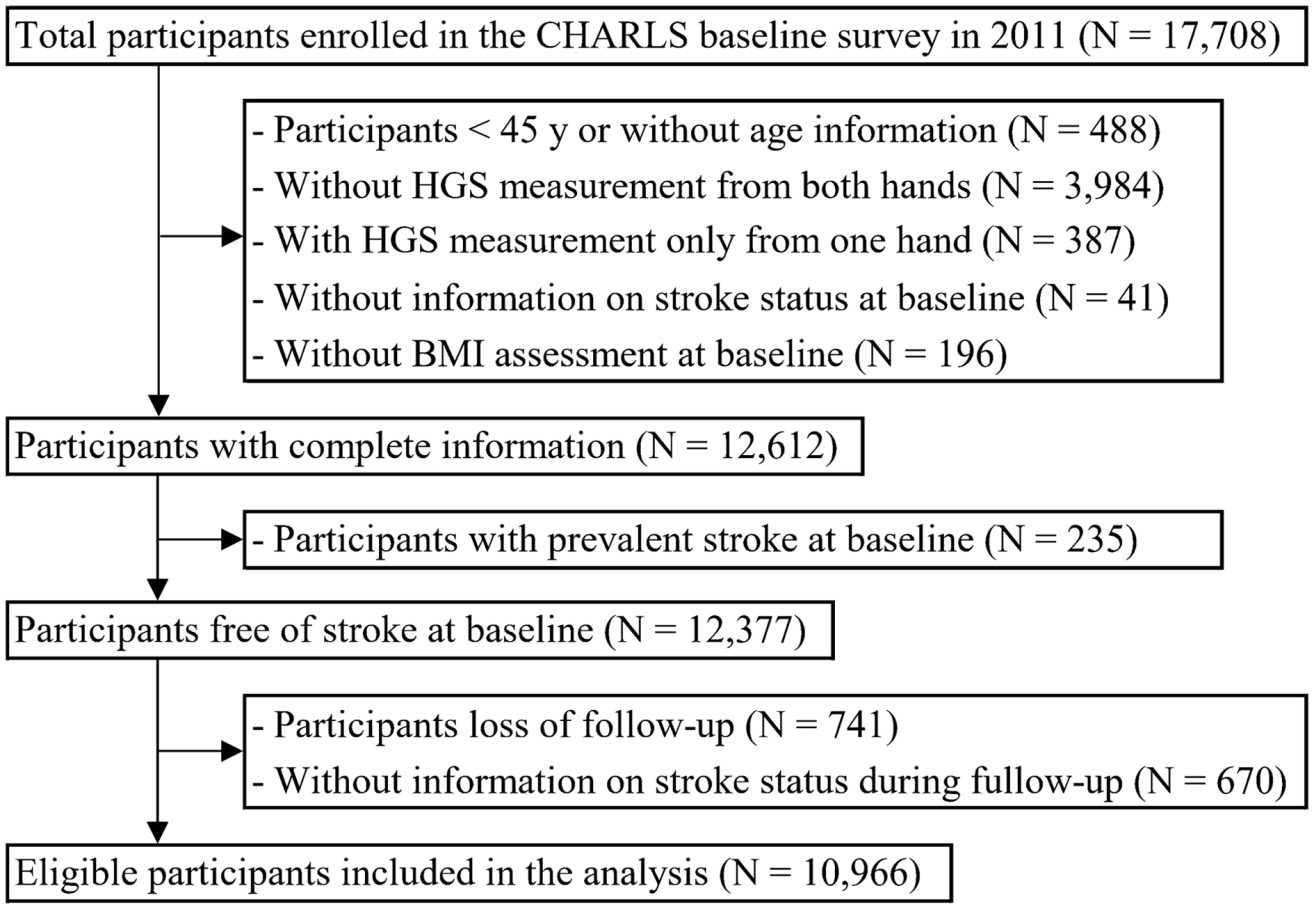
Flowchart of participant selection. BMI, body mass index; HGS, handgrip strength.

In this cohort study, we used data from baseline, 2013, and 2015 follow-up surveys. At baseline, a total of 17,708 participants were recruited (Figure 1). We excluded participants aged under 45 years or without age information (*N* = 488), participants lacking HGS data from both hands (*N* = 3,984) or having HGS measurement only from one hand (*N* = 387), participants with missing information on stroke status (*N* = 41), or body mass index (BMI) (*N* = 196), and participants with prevalent stroke at the baseline survey (*N* = 235), leaving 12,377 eligible participants free of stroke at baseline. We further excluded 741 participants who were lost to follow-up, and 670 participants who lacked information on stroke status in both 2013 and 2015 CHARLS follow-up assessments. Finally, a total of 10,966 participants were included in the current analysis.

The ethics of CHARLS study was approved by the Biomedical Ethics Review Committee of Peking University (IRB approval number: IRB00001052-11015 and IRB00001052-11014) (24). Each participant has signed a written consent form to join the study.

### Measurement of handgrip strength

HGS in kilograms was assessed by a standardized dynamometer (Yuejian WL-1000, China) (24, 27, 28). If participants had undergone surgery, or experienced swelling, inflammation, severe pain, or injury to one or both hands within the last 6 months, or declined to have their HGS measured, no assessment was taken. During the assessment, each participant was guided by trained interviewers to stand with their shoulder in a neutral position; if participants were unable to stand without assistance, a sitting position was allowed. They were then instructed to hold the dynamometer with their elbow flexed at a 90° angle and squeeze the handle with their maximum effort for a few seconds. The measurement was repeated twice for each hand.

To define weak HGS, the maximum value of HGS from both hands was used. According to the recommendation of European Working Group on Sarcopenia in Older People (EWGSOP), participants were first stratified into quartiles based on BMI in males and females, respectively. Then, participants with the lowest 20% of HGS in each stratum were defined as having weak HGS. In the current analysis, the cut-off values for weak HGS in females were 19.0, 21.0, 22.0, and 22.5 kg for those with a BMI ≤21.2, 21.3–23.6, 23.7–26.3, and > 26.3 kg/m^2^, respectively. The cut-off values for weak HGS in males were 29.0, 32.0, 34.0, and 35.0 kg for those with a BMI ≤20.4, 20.5–22.4, 22.5–25.0, and > 25.0 kg/m^2^, respectively.

According to the “10% rule,” HGS of the dominant hand is approximately 10% higher than that of the non-dominant hand (13). Therefore, if the HGS ratio of both hands was over 1.1 or below 0.9, the participant was defined as having asymmetric HGS; otherwise, HGS was defined as symmetric.

All participants were further categorized into four groups according to HGS weakness and asymmetry, i.e., no weakness and asymmetry, asymmetry alone, weakness alone, and both weakness and asymmetry.

### Measurement of stroke

Self-reported stroke status was assessed by the question “Have you been diagnosed with stroke by a doctor?” (29). Answers to this question were “yes” or “no.” A participant was considered as having new-onset stroke if he/she has reported a negative answer at baseline and a positive answer at any of the 2013 and 2015 follow-up surveys. The onset time for stroke was identified as the age at which the stroke was first diagnosed. In cases where this information was absent, we used the midpoint between the most recent wave indicating a stroke diagnosis and the preceding wave when the participant was free of stroke to determine the stroke onset time.

### Covariates

Information on age, sex, educational background, marital status, area of residence, smoking and drinking status were collected by trained investigators through face-to-face interviews. Educational background was categorized into three groups: (1) illiterate or without formal education, (2) primary school, and (3) middle school or above. Marital status was classified as married/cohabitated and unmarried groups. Area of residence was divided into rural and urban areas. Ethnicity was classified into Han ethnicity and other ethnic minorities, as the Han ethnicity is the most populous ethnic group in China. Smoking and drinking status were categorized as current users versus non-current users.

BMI was calculated as weight (kg) divided by the square of height (m^2^). In the Chinese population, a BMI of 28 kg/m^2^ or above was defined as obesity according to the recommendations of Working Group on Obesity in China (30, 31). BP was measured by a digital sphygmomanometer (Omron™ HEM-7200 Monitor, Dalian, China) (24). Glycosylated hemoglobin (HbA1c) and plasma glucose levels were assessed using the affinity high-performance liquid chromatography (HPLC) and the enzymatic colorimetric test, respectively. Physician-diagnosed diabetes mellitus, hypertension, dyslipidemia, and heart diseases were self-reported by each participant. Hypertension was ascertained if one or more of the following criteria was met: (1) with physician-diagnosed hypertension, (2) a mean systolic BP ≥ 140 mmHg, (3) a mean diastolic BP ≥ 90 mmHg, and (4) on anti-hypertensive drugs (32, 33). According to the American Diabetes Association criteria, a participant was defined as having diabetes mellitus if he/she had random plasma glucose ≥11.1 mmol/L, and/or fasting plasma glucose ≥7.0 mmol/L, and/or HbA1c ≥ 6.5%, and/or with self-reported physician-diagnosed diabetes mellitus, and/or on glucose-lowering drugs or insulin treatment (34–36).

### Statistical analysis

Descriptive statistics were expressed as mean ± standard deviation (SD) for continuous data and number (percentage) for categorical data. Characteristics across different HGS weakness and asymmetry groups were compared using one-way analysis of variance (ANOVA) test for continuous data and χ^2^ test for categorical variables. Cox proportional hazards regression analyses were used to examine the association between different HGS weakness and asymmetry status and the risk of new-onset stroke. Participants with no weakness and asymmetry was treated as the reference group in all models. The association was first assessed in a crude model. The adjusted model was controlled for age, sex, educational background, marital status, area of residence, current smoking and drinking status, BMI (not adjusted in weak HGS-related analysis), diabetes mellitus, hypertension, dyslipidemia, and heart diseases, according to previous similar studies (5, 22, 37). The results were presented as hazard ratio (HR) with 95% confidence interval (CI).

Three sensitivity analyses were further conducted to test the robustness of the findings: (1) we examined the associations in different age groups, i.e., <60 years and ≥ 60 years, (2) we excluded participants who developed stroke within the first 2 years of follow-up to avoid reverse causality, and (3) we defined asymmetric HGS if the HGS ratio of both hands was over 1.2 or below 0.8 to test the robustness of the findings.

Data analyses were performed with Stata/SE 15.1 (Stata-Corp, College Station, TX, United States). All tests were two-sided and a value of *p* <0.05 was considered to be statistically significant.

## Results

**Figure 2:**
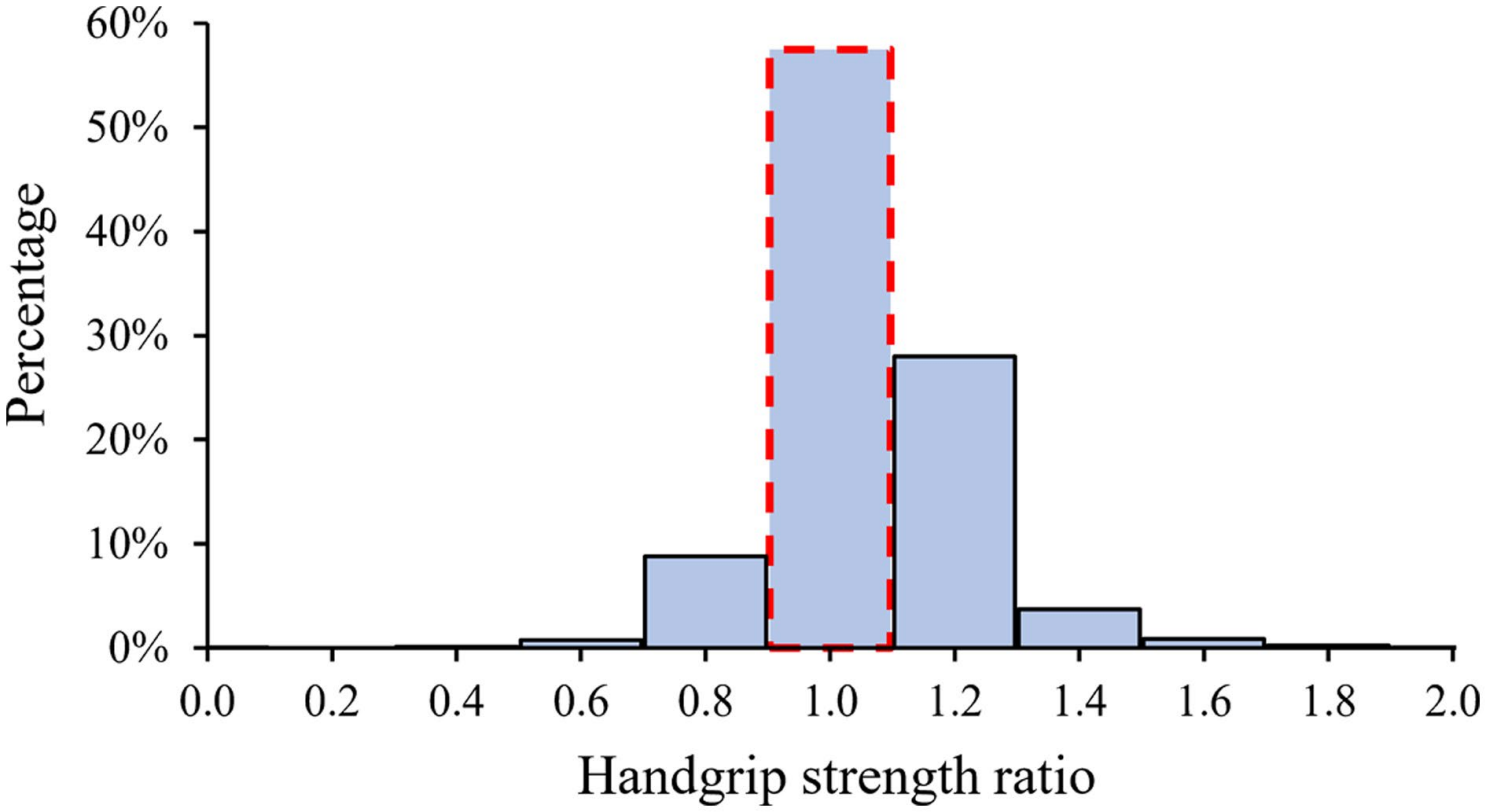
Histogram of HGS ratio. Participants with a HGS ratio over 1.1 or below 0.9 were defined as having asymmetric HGS; otherwise, symmetric HGS was defined. HGS, handgrip strength.

Of the 10,966 participants aged 45 years or older, 5,781 (52.7%) were females and the mean age was 59.2 ± 9.4 years at baseline. Figure 2 depicts a histogram of the HGS ratio among participants, showing that the majority (58.7%) had symmetric HGS, i.e., HGS ratio between 0.9 and 1.1. The number of participants with non-weak and symmetric HGS, asymmetric HGS alone, weak HGS alone, and both weak and asymmetric HGS were 5,232 (47.7%), 3,443 (31.4%), 1,205 (11.0%), and 1,086 (9.9%), respectively (Table 1). Compared to participants without HGS weakness or asymmetry, those with both weak and asymmetric HGS were older, more likely to be females, unmarried, less educated, less obese; and they were also more likely to have higher levels of systolic BP and glucose profiles, and with higher prevalence of self-reported chronic diseases, including diabetes mellitus, hypertension, dyslipidemia, and heart diseases.

**Table 1:** Comparison of baseline characteristics by HGS weakness and asymmetry.

	No weakness and asymmetry	Asymmetry alone	Weakness alone	Both weakness and asymmetry	*p* value
*N* (%)	5,232 (47.7%)	3,443 (31.4%)	1,205 (11.0%)	1,086 (9.9%)	
Demographic and lifestyle factors					
Mean age (years)	57.4 (8.4)	58.0 (8.7)	65.0 (9.5)	65.6 (10.3)	<0.001
Female, *n* (%)	2,646 (50.6%)	1922 (55.8%)	592 (49.1%)	621 (57.2%)	<0.001
Educational background, *n* (%)					<0.001
Illiterate/no formal education	2,226 (42.6%)	1,529 (44.4%)	712 (59.1%)	660 (60.9%)	
Primary school	1,193 (22.8%)	781 (22.7%)	263 (21.8%)	225 (20.8%)	
Middle school or above	1,812 (34.6%)	1,133 (32.9%)	230 (19.1%)	199 (18.4%)	
Area of residence, *n* (%)					0.328
Urban	3,375 (64.5%)	2,203 (64.0%)	804 (66.7%)	713 (65.7%)	
Rural	1,857 (35.5%)	1,240 (36.0%)	401 (33.3%)	373 (34.3%)	
Marital status, *n* (%)					<0.001
Unmarried/cohabitated	478 (9.1%)	377 (10.9%)	238 (19.8%)	234 (21.5%)	
Married	4,754 (90.9%)	3,066 (89.1%)	967 (80.2%)	852 (78.5%)	
Ethnicity, *n* (%)					0.168
Han ethnicity	4,818 (92.1%)	3,145 (91.4%)	1,111 (92.3%)	1,015(93.5%)	
Ethnic minorities	413 (7.9%)	296 (8.6%)	93 (7.7%)	71 (6.5%)	
Current smoker, *n* (%)	1,713 (32.9%)	1,031 (30.0%)	365 (30.4%)	283 (26.3%)	<0.001
Current drinker, *n* (%)	1,922 (36.8%)	1,101 (32.0%)	356 (29.5%)	259 (23.9%)	<0.001
Clinical / biochemical measures					
BMI (kg/m^2^)	23.5 (3.7)	23.5 (3.9)	23.5 (4.2)	23.2 (4.0)	0.040
Obesity, *n* (%)	589 (11.3%)	410 (11.9%)	139 (11.5%)	96 (8.8%)	0.048
Systolic BP (mmHg)	129.5 (20.5)	130.0 (21.0)	134.3 (23.1)	133.8 (23.7)	<0.001
Diastolic BP (mmHg)	76.0 (12.1)	76.2 (12.2)	75.7 (12.7)	75.1 (12.0)	0.053
Glucose (mmol/L)	6.1 (1.9)	6.0 (1.8)	6.4 (2.6)	6.4 (2.6)	<0.001
HbA1c (%)	5.2 (0.8)	5.2 (0.7)	5.4 (1.0)	5.4 (1.0)	<0.001
Total cholesterol (mmol/L)	5.0 (1.0)	5.1 (1.0)	5.0 (1.0)	5.0 (1.1)	0.051
Triglycerides (mmol/L)	1.5 (1.3)	1.5 (1.2)	1.5 (1.3)	1.5 (1.1)	0.456
HDL-cholesterol (mmol/L)	1.3 (0.4)	1.3 (0.4)	1.3 (0.4)	1.3 (0.4)	0.127
LDL-cholesterol (mmol/L)	3.0 (0.9)	3.1 (0.9)	3.0 (0.9)	3.0 (0.9)	0.038
Disease history					
Diabetes mellitus, *n* (%)	607 (11.6%)	386 (11.2%)	199 (16.5%)	169 (15.6%)	<0.001
Hypertension, *n* (%)	1,926 (36.8%)	1,343 (39.0%)	586 (48.6%)	534 (49.2%)	<0.001
Dyslipidaemia, *n* (%)	399 (7.8%)	312 (9.2%)	118 (10.0%)	97 (9.2%)	0.019
Heart diseases, *n* (%)	504 (9.7%)	386 (11.3%)	151 (12.6%)	183 (16.9%)	<0.001

BMI, body mass index; BP, blood pressure; HbA1c, glycated hemoglobin A1c; HDL, high-density lipoprotein; HGS, handgrip strength; LDL, low-density lipoprotein; SD, standard deviation.

Continuous data were reported as mean (SD) and categorical data were reported as frequency (percentage).

During 4 years of follow-up, there were 262 (2.39%) new-onset stroke cases. Compared to non-weak HGS group, those with HGS weakness were more likely to develop stroke (2.01% versus 3.84%, *p* < 0.001) (Table 2). In contrast, the incidence of stroke was comparable between participants with or without asymmetric HGS (2.72% versus 2.16%, *p* = 0.060). When weakness and asymmetry of HGS were taken into consideration at the same time, we found that participants with both conditions had the highest incidence rate of stroke compared to the other three groups (*p* < 0.001).

**Table 2:** Associations of HGS weakness and asymmetry with the risk of new-onset stroke.

	Stroke incidence	Crude model		Adjusted model^a^	
		HR (95%CI)	*p* value	HR (95%CI)	*p* value
HGS weakness					
No weakness	2.01%	Ref	–	Ref	–
Weak HGS	3.84%	1.95 (1.51, 2.51)	<0.001	1.46 (1.10, 1.93)	0.009
HGS asymmetry^b^					
No asymmetry	2.16%	Ref		Ref	
Asymmetry HGS	2.72%	1.26 (0.99, 1.61)	0.060	1.21 (0.94, 1.55)	0.138
HGS weakness and asymmetry					
No weakness and asymmetry	1.93%	Ref		Ref	
Asymmetry only	2.12%	1.10 (0.81, 1.49)	0.534	1.09 (0.80, 1.48)	0.579
Weakness only	3.15%	1.66 (1.14, 2.41)	0.008	1.27 (0.86, 1.88)	0.236
Both weakness and asymmetry	4.60%	2.43 (1.73, 3.41)	<0.001	1.80 (1.24, 2.60)	0.002

HGS, handgrip strength; HR, hazard ratio; CI, confidence interval.

a Adjusted for age, sex, educational background, marital status, area of residence, current smoking and drinking status, diabetes mellitus, hypertension, dyslipidaemia, and heart diseases.

b When the exposure was HGS asymmetry, BMI was also adjusted.

The association of baseline HGS weakness and asymmetry with the risk of new-onset stroke was presented in Table 2. In the crude model, weakness was associated with 1.95 times (95%CI: 1.51–2.51) greater risk of future stroke attack, whereas asymmetry was not significantly associated with the risk of stroke (HR: 1.26, 95%CI: 0.99–1.61). The findings were consistent in fully adjusted models. When HGS weakness and asymmetry were considered at the same time, using non-weak and symmetric HGS as reference, individuals with both weak and asymmetric HGS tended to have elevated risk of stroke (adjusted HR: 1.80, 95%CI: 1.24–2.60) compared to those with weakness alone (adjusted HR: 1.09, 95%CI: 0.80–1.48) or asymmetry alone (adjusted HR: 1.27, 95%CI: 0.86–1.88), albeit the confidence intervals were generally overlapped. Subgroup analysis by age showed similar associations in participants aged ≥60 years, but not in their counterparts who were younger than 60 years (Supplementary Table S1). Consistent results were also found after excluding incident stroke in 2013 CHARLS survey (Supplementary Table S2) or using a different criterion for asymmetric HGS (Supplementary Figure S1).

## Discussion

The present study reported the individual and joint associations of HGS weakness and asymmetry with new-onset stroke among middle-aged and older Chinese in a cohort setting. We demonstrated that individuals with both weak and asymmetric HGS had a tendency of increased risk of new-onset stroke, compared to those with normal HGS, or with either weak or asymmetric HGS alone. Our findings highlighted the importance of examining HGS asymmetry alongside its weakness to improve the risk-stratification and target prevention of stroke.

The significant association between HGS weakness and incident stroke has been demonstrated by several previous studies (5, 22, 38). For example, the Prospective Urban Rural Epidemiology (PURE) study with data from 17 countries has reported a 9% increased risk of incident stroke associated with every 5 kg reduction in HGS (5). A cohort study of over 280,000 participants from UK biobank has also revealed that weak HGS was linked to a higher hazard for both ischemic and hemorrhagic stroke (38). Moreover, a prospective cohort study using data from CHARLS has reported that weakness and declines in HGS were associated with stroke incidence in middle-aged and older Chinese (22). In accord with above-mentioned studies, we also demonstrated that HGS weakness was associated with increased risk of stroke.

The exact mechanisms underlying the association between HGS weakness and risk of stroke are not fully understood. Skeletal muscle is a major organ of energy metabolism, and when muscle mass is reduced, the uptake of glucose is decreased accordingly. This reduction can subsequently lead to insulin resistance (15), a well-established risk factor of stroke (19). Furthermore, previous studies have demonstrated that HGS weakness was associated with increased level of inflammatory factors (18), such as C-reactive protein and interleukin-6, which in turn could contribute to a heightened vulnerability to stroke incidence (20). In addition, weak HGS might be a product of long-term unhealthy lifestyles and imbalanced nutrition (39, 40). These factors are associated with an increased risk of stroke, irrespective of age (41, 42). Therefore, the significant association between HGS weakness and new-onset stroke appears to be physiologically plausible.

Compared to HGS weakness, the association between HGS asymmetry and health outcomes has been underexplored. A cohort analysis using data from CHARLS has demonstrated that individuals with asymmetric HGS had increased hazard of neurodegenerative disorders during the 4 years follow-up, whereas HGS weakness was not an independent contributor to the outcome (10). Another longitudinal study with over 18,000 American adults aged 50 years or above has shown that HGS asymmetry and weakness were independently associated with increased risk of morbidity accumulation, while the odds were even greater in individuals with co-occurrence of the two conditions (11). Similar findings regarding the combined effect of HGS asymmetry and weakness have also been reported on incident functional disabilities (13, 14), low cognitive function (12), and depression (43). Our study further extends the knowledge of previous studies by demonstrating an augmented risk of stroke in individuals with both weak and asymmetric HGS, albeit no significant association was observed when examining the individual association of HGS asymmetry with new-onset stroke. The exact mechanism underlying the increased risk estimates of stroke in participants with both weak and asymmetric HGS remains unclear. Strength asymmetry between limbs might be a precursor of declines in overall strength capacity, and the co-occurrence of HGS weakness and asymmetry might represent a more severe muscle dysfunction than either condition alone (11). This may help explain the exaggerated risk of stroke in participants with both weak and asymmetric HGS. As such, our study further supports the combined assessments of HGS weakness and asymmetry in health screening to identify high-risk individuals for target prevention of stroke.

Subgroup analysis by age further showed that the associations remained consistent in adults aged ≥60 years, while such associations became statistically non-significant in individuals below 60 years. The mechanisms driving this variation are yet to be fully elucidated. In our study sample, younger adults aged <60 years generally achieved higher educational levels. This could potentially render them more perceptive and responsive to weak HGS, subsequently reducing their stroke risk. In addition, middle-aged individuals usually maintain a more active lifestyle compared to their older counterparts. This heightened physical engagement might mitigate the associations between HGS and stroke susceptibility in the younger population. Our research underscores the importance of HGS monitoring and intervention, particularly in the older population.

### Strengths and limitations

The major strength of our study is the cohort design with a nationwide sample using standardized protocols. We not only investigated the individual impact of HGS asymmetry and weakness on stroke incidence, but also the combined impact. However, there are still some limitations that deserve further discussion. First, stroke was defined based on self-report of a physician’s diagnosis, which might cause potential recall bias and misclassification of stroke. Nevertheless, chronic diseases reported by participants has been demonstrated to be reliable compared with information extracted from medical record (44, 45). In addition, in longitudinal studies, the bias from such misclassification is often non-differential with respect to stroke outcome events, thus biasing the measure of association towards the null. Consequently, our results were likely more conservative than the true association. Second, since brain images were not applied to determine history of stroke at baseline, we could not rule out the possibility of minor stroke or transient ischemic attack (TIA) without typical symptoms or medical diagnosis. Nevertheless, it is usually not feasible to do so for every participant in large epidemiological study. The sensitivity analysis excluding incident stroke cases in the first 2 years of follow-up also revealed augmented risk of stroke in participants with both weak and asymmetric HGS, suggesting the robustness of our findings. Third, there was no information regarding the types of stroke attack. Therefore, we were unable to differentiate whether HGS weakness and asymmetry had different associations with ischemic and hemorrhagic stroke. Fourth, although many confounders have been controlled in the statistical models, we could not rule out potential residual confounding effects of other factors that were not captured in the present study, such as family history of stroke or dietary pattern and quality (46–49). In addition, although physical activity is recognized as an important risk factor for stroke (50), it was not included in the analysis due to the lack of data for 58.3% of the included participants. Furthermore, the modified International Physical Activity Questionnaire (IPAQ)-short form used in CHARLS adopted categorical choices to collect participant’s time spent on different intensities of physical activity, which might lead to imprecise estimates of energy expenditure and physical activity levels. Therefore, we did not consider this factor in the current study. Future research should further investigate whether physical activity could alter the associations observed in the present study.

## Conclusion

Our study demonstrated that HGS weakness in combination with its asymmetry was associated with an increased risk of new-onset stroke in Chinese middle-aged and older adults. The risk estimates tended to be larger than that observed in individuals with normal HGS, or those only with weak HGS or asymmetric HGS. Our findings could provide valuable insights into early identification and intervention for stroke development. Targeting individuals with both weak and asymmetric HGS might have substantial benefits in lowering the risk of stroke. However, future randomized controlled trials are needed to confirm the conclusion.

## Data availability statement

Publicly available datasets were analyzed in this study. This data can be found in a public, open access repository, and can be accessed at China Health and Retirement Longitudinal Study (CHARLS) http://charls.pku.edu.cn/index/en.html. Requests to access the datasets should be directed to https://charls.pku.edu.cn/en/. The original contributions presented in the study are included in the article/supplementary material, further inquiries can be directed to the corresponding author/s.

## Ethics statement

The ethics of CHARLS was approved by the Biomedical Ethics Review Committee of Peking University (IRB approval number: IRB00001052-11015 and IRB00001052-11014) in accordance with the local legislation and institutional requirements. The participants provided their written informed consent to participate in CHARLS. The current study is a secondary data analysis based on the public data of CHARLS, which is exempt from further ethical approval according to relevant regulations.

## Author contributions

BC and VG: conceptualization and supervision. VG: data curation. YZ and VG: formal analysis, funding acquisition, and writing – original draft. YZ, WC, BC, JL, and VG: methodology. WC, BC, LL, and VG: validation. YZ, WC, BC, LL, JL, and VG: writing – review and editing. All authors contributed to the article and approved the submitted version.
