# Supplementary material for: Associations of handgrip weakness and asymmetry with new-onset stroke in Chinese middle-aged and older adults: a cohort study
Source: Front Public Health. 2023 Oct 16;11:1251262. doi: 10.3389/fpubh.2023.1251262 (PMC10615130; doi:10.3389/fpubh.2023.1251262)
Supplement: Supplementary file 1 [file Table_1.DOCX]

Associations of handgrip weakness and asymmetry with new-onset stroke in Chinese middle-aged and older adults: a cohort study

Yuying Zhang, Weiqing Chen, Bing Cao*, Li Lin, Jinghua Li, Vivian Yawei Guo*

*** Corresponding authors:**

Bing Cao,

Departments of Neurosurgery, Neuropsychiatry and Behavioral Sciences, Stanford Neuroscience Institute, Stanford University School of Medicine, Stanford, CA, USA

Tel: +1-650-725-2530;

Email: [bingcao@stanford.edu](mailto:bingcao@stanford.edu)

Vivian Yawei Guo,

Department of Epidemiology, School of Public Health, Sun Yat-sen University, 74 Zhongshan Second Road, Guangzhou, Guangdong 510080, China.

Tel: +86-20-87331605;

Email: [guoyw23@mail.sysu.edu.cn](mailto:guoyw23@mail.sysu.edu.cn)

**Supplementary Table 1. Associations of HGS weakness and asymmetry with the risk of new-onset stroke by different age groups.**

|  | **Age<60 years^1^** | |  | **Age≥60years^1^** | |
| --- | --- | --- | --- | --- | --- |
|  | **HR (95%CI)** | **p-value** |  | **HR (95%CI)** | **p-value** |
| **HGS weakness** |  |  |  |  |  |
| No weakness | Ref | - |  | Ref | - |
| Weakness | 1.20 (0.70, 2.05) | 0.513 |  | 1.67 (1.20, 2.32) | 0.002 |
| **HGS asymmetry**^2^ |  |  |  |  |  |
| No asymmetry | Ref |  |  | Ref |  |
| Asymmetry | 1.34 (0.91, 1.98) | 0.136 |  | 1.15 (0.83, 1.59) | 0.411 |
| **HGS weakness and asymmetry** |  |  |  |  |  |
| No weakness and asymmetry | Ref |  |  | Ref |  |
| Asymmetry only | 1.47 (0.96, 2.24) | 0.074 |  | 0.81 (0.51, 1.27) | 0.356 |
| Weakness only | 1.58 (0.77, 3.24) | 0.213 |  | 1.19 (0.75, 1.90) | 0.451 |
| Both weakness and asymmetry | 1.25 (0.56, 2.81) | 0.584 |  | 1.94 (1.27, 2.96) | 0.002 |

Abbreviations: HGS, handgrip strength; HR, hazard ratio; CI, confidence interval.

^1^ Adjusted for sex, educational background, marital status, area of residence, current smoking and drinking status, diabetes mellitus, hypertension, dyslipidaemia, and heart diseases.

^2^ When the exposure was HGS asymmetry, BMI was also adjusted.

**Supplementary Table 2. Associations of HGS weakness and asymmetry with the risk of new-onset stroke, excluding incident cases in 2013 CHARLS.**

|  | **Crude model** | |  | **Adjusted model^1^** | |
| --- | --- | --- | --- | --- | --- |
|  | **HR (95%CI)** | **p-value** |  | **HR (95%CI)** | **p-value** |
| **HGS weakness** |  |  |  |  |  |
| No weakness | Ref | - |  | Ref | - |
| Weakness | 2.29 (1.54, 3.42) | <0.001 |  | 1.88 (1.23, 2.87) | 0.004 |
| **HGS asymmetry^2^** |  |  |  |  |  |
| No asymmetry | Ref |  |  | Ref |  |
| Asymmetry | 1.35 (0.92, 1.99) | 0.127 |  | 1.30 (0.87, 1.94) | 0.197 |
| **HGS weakness and asymmetry** |  |  |  |  |  |
| No weakness and asymmetry | Ref |  |  | Ref |  |
| Asymmetry only | 0.95 (0.58, 1.57) | 0.851 |  | 0.95 (0.57, 1.59) | 0.849 |
| Weakness only | 1.45 (0.78, 2.72) | 0.240 |  | 1.24 (0.65, 2.34) | 0.518 |
| Both weakness and asymmetry | 3.15 (1.91, 5.19) | <0.001 |  | 2.55 (1.50, 4.36) | 0.001 |

Abbreviations: HGS, handgrip strength; HR, hazard ratio; CI, confidence interval.

^1^ Adjusted for age, sex, educational background, marital status, area of residence, current smoking and drinking status, diabetes mellitus, hypertension, dyslipidaemia, and heart diseases.

^2^ When the exposure was HGS asymmetry, BMI was also adjusted.


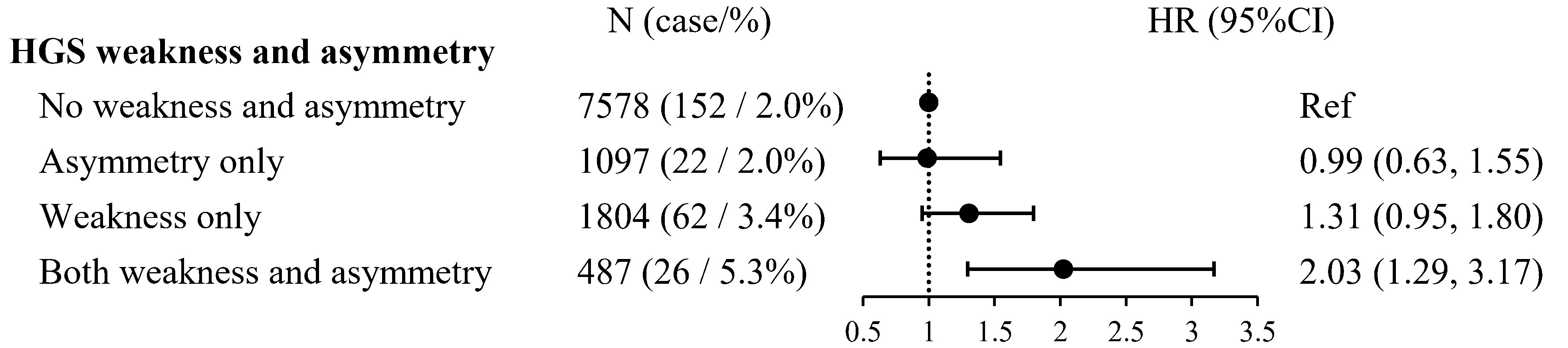


**Supplementary Fig. 1 Associations of HGS weakness and asymmetry with the risk of new-onset stroke, when HGS asymmetry was defined as an HGS ratio of both hands over 1.2 or below 0.8.**

HGS: handgrip strength; HR: hazard ratio; CI: confidence interval.

^a^ Adjusted for age, sex, educational background, marital status, area of residence, current smoking and drinking status, diabetes mellitus, hypertension, dyslipidaemia, and heart diseases.
